# Evaluation of compressive and adhesive strengths of a hybrid ceramic and 5Y‐PSZ zirconia cemented with three different materials

**DOI:** 10.1111/eos.70017

**Published:** 2025-05-27

**Authors:** Letícia Moreschi, João Paulo De Carli, Marciele Cristiane Spanemberg Fuhr, Renan Brandenburg dos Santos, Paulo Renato Pulga da Silva, Pedro Henrique Corazza

**Affiliations:** ^1^ Graduate Program in Dentistry, University of Passo Fundo Passo Fundo Rio Grande do Sul Brazil; ^2^ School of Dentistry University of Passo Fundo Passo Fundo Rio Grande do Sul Brazil

**Keywords:** cementation, ceramics, dental materials

## Abstract

This study aimed to evaluate the compressive and adhesive strengths of ceramic restorations (PICN and 5Y‐PSZ zirconia) cemented with one of three types of cements: conventional glass ionomer cement (GI), resin‐modified glass ionomer cement (RM‐GI), and self‐adhesive resin cement (R). Ceramic specimens (5Y‐PSZ, *n* = 72; PICN, *n* = 60) were prepared for testing after cementation onto glass fiber‐reinforced resin‐based composite tooth analogs. The 5Y‐PSZ specimens underwent tribochemical silica coating before cementation. Specimens intended for compressive strength evaluation were mechanically cycled (500,000 cycles) before testing. A microtensile bond strength test was used to evaluate adhesive strength, with specimens sectioned into microbars (1 mm^2^ cross‐section) and loaded until fracture. If pre‐test failures occurred, bond strength was assessed using microshear tests. PICN restorations showed significantly higher compressive strength than 5Y‐PSZ. For PICN, self‐adhesive resin cement yielded the highest compressive strength. For adhesive strength, PICN restorations cemented with self‐adhesive or RM‐GI cements outperformed GI. The 5Y‐PSZ+R combination showed the highest microshear bond strength, superior to 5Y‐PSZ+RM‐GI and 5Y‐PSZ+GI. Resin cements showed higher compressive and adhesive strengths for PICN than glass ionomer‐based cements. For 5Y‐PSZ, the cement type did not significantly affect compressive strength. Overall, PICN restorations outperformed 5Y‐PSZ in compressive and adhesive strengths.

## INTRODUCTION

Ceramic restorations are widely used in dentistry to replicate the characteristics of dental tissues, making them a preferred choice among both professionals and patients [1]. In order to achieve aesthetic and mechanical properties similar to natural teeth, various types of dental ceramics have been introduced, including feldspathic, leucite‐reinforced, lithium disilicate, fluorapatite glass‐ceramics, hybrid ceramics known as polymer‐infiltrated ceramic‐network (PICN), and zirconia [2].

The PICN has been employed in dental restorations due to its excellent elastic modulus, which is close to that of natural teeth, and its biocompatibility [3]. Della Bona et al. [4] demonstrated through microstructural analyses that PICN is a hybrid material consisting of interconnected networks: a dominant ceramic phase and a polymer phase, resulting in properties that lie between highly filled composites and ceramics. Zirconia has also been extensively studied as an efficient restorative material. With advancements in the dental field, third‐generation partially stabilized zirconia (PSZ), such as 4Y‐PSZ and 5Y‐PSZ, have been developed to address the esthetic challenges associated with first‐generation zirconia [5]. However, literature suggests that their strength is lower compared to traditional first‐generation zirconia [6]. One limiting factor for the use of zirconia is the low bonding potential between zirconia restorations, cement, and the dental structure, as conventional cementation protocols for glass ceramics do not apply to zirconia‐based ceramics [7].

The longevity of ceramic restorations is influenced by the choice of cementing agent [8]. Resin cements were developed to provide the necessary physical and mechanical properties for the successful cementation of all‐ceramic restorations [9]. These cements can be categorized into conventional resin cements, which require an adhesive system due to their lack of inherent bonding to the dental structure, and self‐adhesive resin cements, which do not require prior adhesive treatment of the dental substrate [10]. Compared to other cements, resin cements are distinguished by their mechanical strength, biocompatibility, and ability to bond to single‐unit prostheses, cores, and short crowns, making them widely used with ceramic restorations [11]. Additionally, they display an average flexural strength that is significantly higher than that of other cements, such as glass ionomer (66.7 MPa vs. 11.65 MPa, respectively) [12].

Glass ionomer cement is also an option for cementation, offering beneficial strength and retention characteristics, with low solubility and clinical longevity [13]. It exhibits viscoelastic properties that are more favorable for maintaining bond integrity compared to stiffer resin cements during polymerization shrinkage, and demonstrates hygroscopic expansion post‐maturation, which compensates for its initial setting contraction, thereby maintaining a more stable dentin/cement interface [14]. With the evolution of dental cements, resin‐modified glass ionomer cements have emerged. These cements are hybrid, consisting of traditional glass ionomer with the addition of light‐curable resin, thus offering intermediate properties of their two elements. However, they present superior characteristics to conventional glass ionomer cements, such as a flexural strength value of 41.07 MPa [12]. They can combine the benefits of both materials, such as bonding to dental structure, good aesthetics, fluoride release, and rapid polymerization under light [15].

Given that glass ionomer cement and resin cements share some physical properties despite their distinct nature, comparing their adhesive and compressive performance when used with new restorative materials is clinically relevant. Some studies have evaluated the adhesive performance of cements associated with zirconia and PICN. It has been reported that self‐adhesive resin cements exhibited superior bond strength compared to conventional glass ionomer cements when used with zirconia, particularly after artificial aging protocols [16]. Similarly, resin‐modified glass ionomer cements showed good bond strength with PICN, although resin cements performed better [17]. These findings highlight the importance of cement selection for optimal adhesive performance. Therefore, the aim of this study was to evaluate the compressive strength and adhesive bond strength of two ceramics (PICN and zirconia) cemented with one of three different materials: a resin cement, a glass ionomer cement, and a resin‐modified glass ionomer cement. The hypothesis of the study is that the compressive and adhesive strengths of ceramic restorations will be higher when associated with self‐adhesive resin cements or resin‐modified glass ionomer cements (RM‐GI) than when conventional glass ionomer cement (GI) is used.

## MATERIAL AND METHODS

The ceramics used in this study were a 5Y‐PSZ zirconia (Zircon Fit Plus, Talmax) with 49% translucency, and a polymer‐infiltrated ceramic network (PICN, Vita Enamic, VITA Zahnfabrik). The three cements used were (i) conventional glass ionomer cement (GI)—GC Gold Label 1 Luting & Lining Cement, GC; (ii) resin‐modified glass ionomer cement (RM‐GI)‐ GC Fuji Plus C, GC; and (iii) self‐adhesive resin cement (R)—Relyx U200, 3 M ESPE. Compressive and adhesive strengths were measured after cementing the ceramics onto a dentin analogue made from glass fiber‐reinforced resin‐based composite [16] using the three different cements. For compressive strength, the restoration/cement/dentin assembly was aged by mechanical cycling (500,000 cycles) and loaded in compression until fracture. Adhesive strength was evaluated using microtensile testing. However, the zirconia‐restored assembly experienced pre‐test failures during the preparation of specimens for microtensile testing. Therefore, adhesive strength for this material was evaluated using microshear testing. The sample size calculations for the three tests were based on a pilot study, use of an ANOVA test, a significance level of 5%, a statistical power of 80%, and the maximum expected difference among groups. Accordingly, the estimated sample sizes were 8 for the microshear test, 10 for the compression test, and 17 for the microtensile test. The power of sample size was calculated after running the three tests (compression, microtensile and microshear bond strength) used in the study. The calculated power of sample was 0.92, 0.83 and 0.99 for compression, microtensile and microshear bond strength, respectively. Therefore, the adequacy of the sample size to detect differences in the three tests was confirmed.

### Preparation of specimens

CAD/CAM blocks of the restorative materials (5Y‐PSZ and PICN) were sectioned with a diamond disc under cooling in a metallographic cutter (Struers Minitron, Struers) to obtain blocks with a final size of 12 mm × 12 mm, considering the zirconia shrinkage after sintering. Specimens with 1 mm thickness were prepared for compression tests, and 4 mm thick specimens were prepared for microtensile and microshear tests. All specimens were polished using a polishing machine (Struers Abramin, Struers) with silicon carbide sandpapers (#400, 600, 800, 1200) until the desired thickness was achieved. A total of 72 specimens of 5Y‐PSZ and 60 specimens of PICN were prepared. The dentin analog was similarly obtained by sectioning the glass fiber‐reinforced resin‐based composite cylinder material using a metallographic cutter, resulting in 132 discs with a 12 mm diameter and 4 mm thickness. The dentin analog treatment was standardized across all groups by cleaning the discs with a Robinson brush and prophylactic paste, followed by rinsing with water for 15 s and drying for the same time. The glass fiber‐reinforced resin‐based composite dentin analog exhibits an elastic modulus of 14.7 GPa, a Poisson's ratio of 0.4, and a Knoop hardness of 52.7 GPa, making it a suitable analogue material [18].

### Surface treatments of PICN and 5Y‐PSZ ceramics

The surface of the PICN specimens (*n* = 60) was treated according to the manufacturer's recommendation, with 5% hydrofluoric acid for 60 s, followed by rinsing with water and drying with air jets. The 5Y‐PSZ specimens (*n* = 72) were sintered using the Programat CS2 furnace (Ivoclar). Following the manufacturer's instructions, sintering occurred over 2 h with a temperature ramp‐up rate of 10°C/min, reaching a temperature range of 1450°C to 1550°C, followed by natural cooling. Subsequently, the thicknesses of all 5Y‐PSZ specimens were verified using a digital caliper, allowing a variation of ± 10% from the predetermined thicknesses. The surfaces were then sandblasted with 30 µm tribochemical silica‐coated aluminum oxide particles (CoJetTM Sand, 3 M‐ESPE) for 20 s at a pressure of 24–28 psi, maintaining a working distance of 10 mm.

### Cementation of specimens for compression and microtensile testing

**FIGURE 1:**
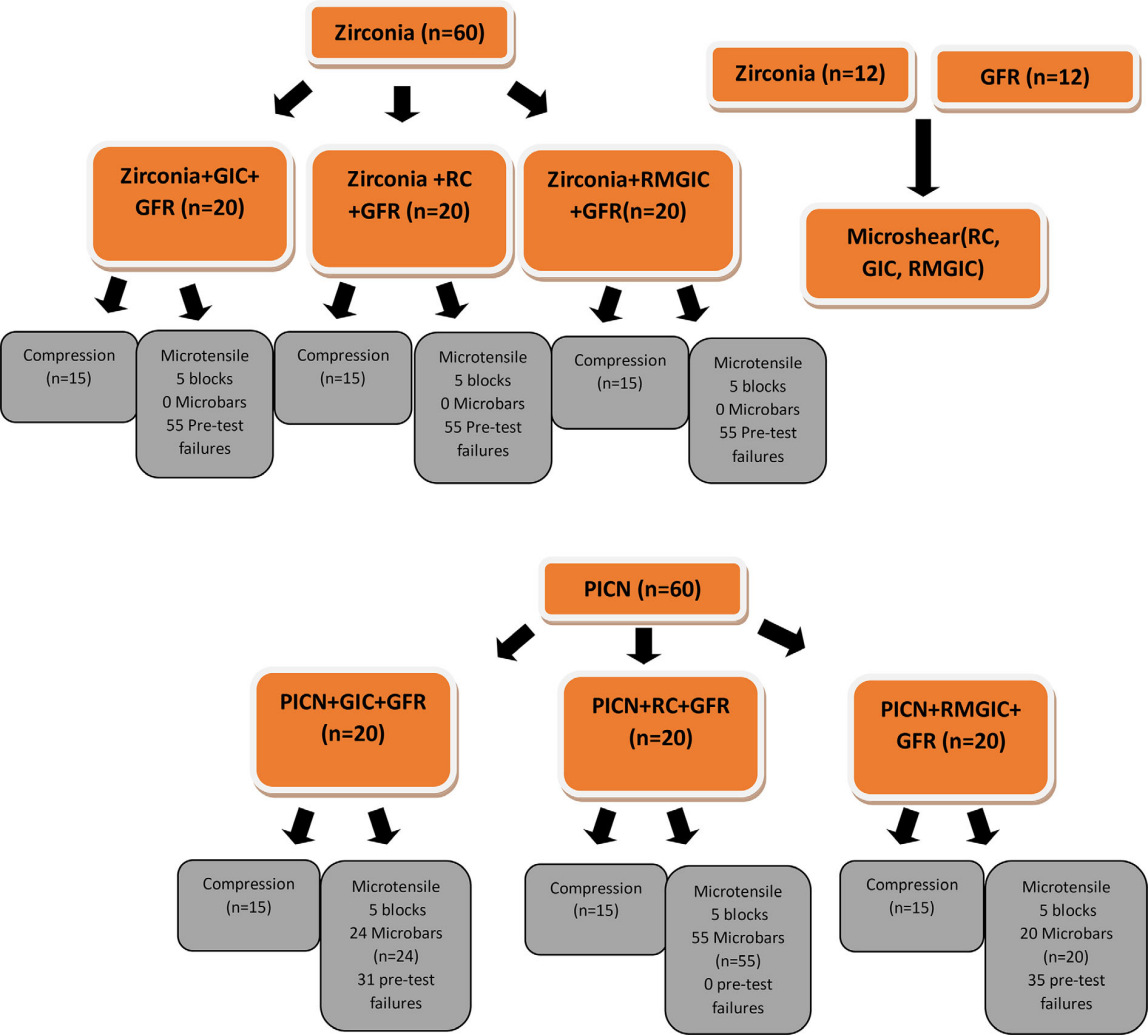
Study flow chart.

A silane agent (RelyX Ceramic Primer, 3 M ESPE) was applied to the surfaces of all 5Y‐PSZ (*n* = 60) and PICN (*n* = 60) specimens using a microbrush, followed by 5 s of air drying. Afterward, cementation was performed according to the experimental protocol with either conventional glass ionomer cement (GI), resin‐modified glass ionomer cement (RM‐GI), or self‐adhesive resin cement (R). Once the surfaces were adequately prepared, cements were dispensed onto a glass slab, mixed until homogeneous, and applied to the surface of each material. Each specimen was individually placed onto the dentin analog, with a standardized load of 750 g applied for 5 min to ensure consistent cement thickness and force. After applying the load, excess cement was removed using a microbrush (FGM), followed by light‐curing in the self‐adhesive resin cement group for 40 s on each surface using a VALO Grand ING (Ultradent) LED curing light with 1400 mW/cm^2^ intensity. This process resulted in 15 specimens per treatment protocol for compression testing and five sets per group for microtensile test, as depicted in the flowchart (Figure 1). In the microtensile test, each cemented block was anticipated to produce approximately 11 micro‐specimens suitable for testing.

### Compression testing

The specimen assemblies subjected to compression testing (*n* = 15/treatment protocol) were mechanically aged to simulate the oral environment using a mechanical cycler (BioPDI). The following parameters were set: a frequency of 2 Hz; a load of 50 N, for 500,000 cycles, equivalent to approximately 2 years of clinical function [19], in distilled water at 37°C, using a flat‐tipped metallic piston (stainless steel) with a 3 mm diameter, centered on the specimen. After mechanical cycling, the specimens were transferred to a universal testing machine (EMIC DL 2000, Instron), equipped with a 5000 N load cell. A compressive load was applied to the center of the specimen at a crosshead speed of 0.5 mm/min using a piston (*E* = 200 GPa) with a flat 3 mm diameter tip. The test was stopped upon audible detection of the first crack by the operator, and the load was recorded in Newtons.

Cracks that lead to failure were classified as radial, when failure originated from the lower surface of the restorative material (intaglio surface); conical, when failure originated from the upper surface of the restorative material (in contact with the loading piston); mixed, when both radial and cone cracks were identified; and catastrophic, when there was a complete failure of the specimen.

### Microtensile testing

Five specimen blocks from each treatment group were sectioned into microbar shape specimens, with a cross‐sectional area at the bonding site of 1 mm^2^, and fixed with cyanoacrylate adhesive (Super Glue, 3 M) onto a guillotine‐shaped metallic device. The microtensile test was conducted on a universal testing machine at a crosshead speed of 0.5 mm/min until fracture occurred, and force values were recorded in Newtons. All pre‐test failures were recorded. As pre‐test failures occurred in all specimens of the 5Y‐PSZ group during the microbars cutting process, adhesion was evaluated using the microshear test. The microbar specimen was considered the experimental unit for the data analysis [20, 21, 22] (Figure 1).

### Microshear test for adhesion of 5Y‐PSZ to dentin analogue

The microshear test was also conducted using a universal testing machine. Slices of 5Y‐PSZ (*n* = 12) and dentin analogue (*n* = 12) were individually fixed in circular tubes using acrylic resin. A silane agent (RelyX Ceramic Primer, 3 M ESPE) was applied to the surfaces of all 5Y‐PSZ and dentin analogue slices using a microbrush, followed by air drying for 5 s. Three silicone cylinders were placed on each surface, creating holes with a diameter of 1 mm and a height of 2 mm, and secured with pink dental wax. Each hole was filled with a different cement, identified on the acrylic with a permanent marker. The same three cements used in the compression and microtensile tests were employed. So, all cylinders were inspected under an optical microscope, and those showing failures or bubbles were excluded from the study. The specimen assemblies were then positioned in a universal testing machine, and a controlled shear force was applied to the bonded interface with a guillotine‐shaped device, at a speed of 0.5 mm/min until failure occurred. A 100 N load cell was used. The bond strength (in MPa) was calculated by dividing the force (N) by the bonded area (mm^2^).

### Data analysis

The compressive load results were analyzed using a two‐way ANOVA using cement (conventional glass ionomer cement [GI], resin‐modified glass ionomer cement [RM‐GI], or self‐adhesive resin cement [R]) and ceramic substrate (5Y‐PSZ or PICN) as the factors, followed by a Tukey test (*α* = 0.05). The microtensile and microshear bond tests generated results that were compared using a one‐way ANOVA and Tukey test (*α* = 0.05), as a comparison between the two different tests was not feasible.

## RESULTS

The results of the compression strength test are described in Table 1. The comparison of results using two‐way ANOVA demonstrated statistical significance for the factors “ceramic substrate” (*p* < 0.001), “cement” (*p* < 0.001), and their interaction (*p* = 0.007). Regardless of the cement used, the groups with PICN as the ceramic substrate achieved higher compression strength values than the groups restored with 5Y‐PSZ. The PICN ceramic substrate bonded with self‐adhesive resin cement (R) achieved the highest compression values, which were statistically significantly superior to the others. The PICN ceramic substrate bonded with GI or RM‐GI presented similar mean values of the compressive bond strength (1992 and 2096, respectively), and these did not differ statistically significantly. There were no statistically significant differences in the mean compression strength values among the three cements when used with 5Y‐PSZ. During mechanical cycling, failures occurred in 2 samples of the PICN+GI group, 2 samples of the 5Y‐PSZ+GI group, 3 samples of the 5Y‐PSZ+RM‐GI group, and 3 samples of the 5Y‐PSZ+R group.

Regarding types of failures, most failures were either mixed or catastrophic. All groups, except the PICN+GI group, exhibited predominantly mixed failures. The PICN+GI group predominantly showed catastrophic failures (77%).

For the microtensile test, each cemented block was expected to yield an average of 11 microbars. For the PICN+R group, there were no pre‐test failures during cutting, so all 55 generated microbars could be tested. In the PICN+GI group, 31 failures occurred during cutting, resulting in 24 microbars for testing. In the PICN+RM‐GI group, 35 microbars were lost during cutting, resulting in 20 microbars being tested. The results of the microtensile test with PICN restorations showed that the PICN+R and PICN+RM‐GI groups did not differ statistically significantly in adhesive strength, whereas the PICN+GI group had statistically lower mean adhesive strength compared to the other two groups (Table 2). Groups involving 5Y‐PSZ zirconia exhibited pre‐test failures during block sectioning in all scenarios. Therefore, adhesion was assessed using the micro shear test, both at the interface with 5Y‐PSZ and at the dentin analogue interface. It was observed that the 5Y‐PSZ+R combination had the greatest micro shear bond strength value, statistically superior to the 5Y‐PSZ+RM‐GI and 5Y‐PSZ+GI groups (Table 2). This was observed at both interfaces: 5Y‐PSZ and dentin analogue.

**TABLE 1:** Mean values (± SD) of the compressive load (in Newtons—N) for the experimental groups defined by Material and Cement used.

				Failures			
Material	Cement	*N*	Mean (*N*) ± SD^a^	Radial	Conical	Mixed	Catastrophic
PICN	R	15	2657.5 ± 408^A^	13.3%	20%	46.7%	20%
	RM‐GI	15	2096.1 ± 315.5^B^	0%	20%	46.7%	33.3%
	GI	13	1992.0 ± 480^B^	0%	0%	23%	77%
5Y‐PSZ	R	12	1400.8 ± 274.6^C^	0%	0%	100%	0%
	RM‐GI	12	1312.7 ± 181.6^C^	0%	0%	66.6%	33.3%
	GI	13	1301.9 ± 216.8^C^	0%	0%	92.3%	7.6%

*Note*: Percentage of each type of failure within each group.

^a^ Different capital letters in the same column indicate statistical difference (Tukey test, *α* = 0.05).

**TABLE 2:** Mean values and standard deviations (SD) of the microtensile bond strength for the PICN.

	PICN Microtensile bond strength (MPa)		5Y‐PSZ and dentin analogue Microshear bond strength (MPa)		
Cement	*n*	Mean (SD)^a^	*n*	Mean (SD)a	Mean (SD)a
				**5Y‐PSZ**	**Dentin analogue**
**R**	55	10.5 (7.9)^A^	12	6.3 (3.5)^A^	7.3 (2.3)^A^
**RM‐GI**	18	13.1 (1.7)^A^	12	2.0 (0.6)^B^	2.9 (1.1)^B^
**GI**	20	5.5 (4.7)^B^	12	1.9 (0.8)^B^	2.4 (0.7)^B^

*Note*: Given is the microshear bond strength for 5Y‐PSZ and dentin analog specimens.

^a^ Different capital letters in the same column indicate statistical difference (Tukey test, *α* = 0.05).

## DISCUSSION

This study aimed to evaluate the compressive and adhesive strength of two ceramics—PICN and 5Y‐PSZ—cemented using three different materials: a resin cement, a glass ionomer cement, and a resin‐modified glass ionomer cement. The results demonstrated that the groups restored with PICN performed better compared to those restored with 5Y‐PSZ. Specifically, PICN showed better results in compressive load tests compared to 5Y‐PSZ zirconia. This is a significant finding considering that the flexural strength of 5Y‐PSZ zirconia is approximately 562 MPa [23], higher than that of PICN, which is typically around 160 MPa [24], for three‐point flexural tests. However, it is known that the strength of third‐generation zirconia is lower when compared to traditional zirconia‐based restorations [6]. This finding may be justified by the greater adhesion of PICN to the cementing agent, creating a single body with higher compressive strength. This was evident in the preparation of specimens for the microtensile test, where the groups restored with 5Y‐PSZ exhibited pre‐test failures in all situations. Additionally, the resin matrix of PICN provides a material elasticity modulus closer to that of the dentin analogue base [4]. Facenda et al. [25] investigated the effect of substrate elastic properties and restorative material on the fatigue resistance of multilayer structures using PICN and an indirect composite resin. They found that restorative materials with lower elastic modulus performed better when bonded to substrates of similarly low modulus. This finding supports the present results, suggesting that the elastic compatibility between PICN and the fiber‐reinforced resin‐based substrate may have contributed to the improved compressive performance. The results of the compression tests performed with translucent zirconia showed no statistically significant differences among the evaluated groups. In a similar study with monolithic 3Y‐TZP zirconia, Nakamura et al. [26] found that resin cements significantly increased compressive strength compared to other cements. The different results obtained in the present study may be due to the restorative material: 3Y‐TZP has higher strength than 5Y‐PSZ and may be more sensitive to bonding agent variations. Differences in experimental design may also contribute to the contrasting results. Berger et al. [27] found that zinc phosphate cement and glass ionomer cement significantly increased 2D and 3D marginal fit discrepancies after cementation, affecting this essential aspect concerning the long‐term clinical success of prosthetic restorations. The findings of Lawson et al. [28] underline the importance of cement type in the strength of dental materials, specifically in zirconia and lithium disilicate, and also observed superior performance with resin cements compared to resin‐modified glass ionomer in the fracture load of traditional yttria‐stabilized zirconia (3Y‐PSZ), translucent yttria‐stabilized zirconia (5Y‐Z), and lithium disilicate crowns. The possible reasons for these discrepancies in results between the present study and the studies of Nakamura et al. [26] and Lawson et al. [28] may be related to methodological issues such as the applied cement. Despite the same classification, there are several commercial brands of cementing agents available in the dental market. In the present study, GC Gold Label 1 Luting & Lining Cement and GC Fuji Plus C from GC were used. According to Magwa et al. [29], the mean flexural strength for the glass ionomer cement (GC Gold Label) and resin‐modified glass ionomer (GC Fuji Plus) is 7.488 MPa and 13.108 MPa, respectively. The authors attribute these differences to the presence of the polymerized resin component, which enhances its ability to withstand flexural loads. Kheur et al. [12] found an average flexural strength of 11.65 MPa for GC Gold Label and 41.07 MPa for GC Fuji Plus. Other factors, such as surface treatment and yttria concentration of the chosen zirconia, may also influence results in different types of tests [30, 31].

In the sectioning of 5Y‐PSZ zirconia for the microtensile test, pre‐test failures were observed in all samples, preventing the test from being conducted and, consequently, the acquisition of results. Such failures suggest that satisfactory adhesion did not occur between the three chosen cements and the cementation surfaces. Therefore, the microshear test was used. The results of zirconia microshear demonstrated that resin cement achieved the best performance compared to glass ionomer‐based cements. Vivek et al. [32], in a study with a similar methodology, compared the shear bond strength of zirconia to dentin using two resin‐based luting cements and a resin‐modified glass ionomer cement. The results showed better adhesion of zirconia to dentin with resin‐based luting cements. On the other hand, Torres et al. [33], in a clinical study, found no significant difference between resin cement and glass ionomer cement in the survival rates of zirconia crowns. Although conventional and resin‐modified glass ionomer cements are commonly employed in cementing zirconia restorations, their efficacy is often compromised by lower bond strength values compared to resin cements. This disparity can be attributed to the lack of chemical adhesion, considered a crucial factor in achieving effective bond strength with zirconia material [34]. These findings may justify the results found in this study, particularly the superior performance of the resin cement in the microshear test and the pre‐test failures that occurred during the microtensile test. For PICN, a difference in compressive strength provided by the three tested cementing agents (R, GI, and RM‐GI) was observed, demonstrating a significantly higher average compressive strength in the R group compared to the other two groups. This result agrees with the study by Vohra et al. [35], who evaluated the compressive strength of lithium disilicate crowns cemented on dentin with a bioactive cement, a resin cement, or a glass ionomer cement, and found that resin cement showed the highest fracture resistance among all groups. Additionally, crowns cemented with glass ionomer cement showed significantly lower failure loads than those cemented with bioactive and resin cements. Saleem et al. [36], testing if the strength parameters greatly influence the selection of luting agent, also verified that resin cement showed significantly higher compressive strengths and diametral tensile strengths among the three tested luting cements (conventional glass ionomer, resin‐modified glass ionomer, and resin cement), while the conventional glass ionomer showed the lowest strength values.

The types of fractures were classified as radial, conical, mixed, or catastrophic. Radial cracks come from the adhesive interface, conical cracks from the external surface (load application region), mixed failures occur when both radial cracks and conical cracks are found, and catastrophic fractures occur when there is a complete failure of the specimen, with material detachment [37]. The most common types of failures found in the three cement groups tested with 5Y‐PSZ zirconia were mixed failures. In a clinical study monitoring the survival of fixed prostheses conducted by Botelho et al. [38], the primary failures were also identified as mixed, with the cement present on both the zirconia‐tooth interface and the abutment teeth. Similarly, Chaar and Kern [39] reported comparable results in a study involving fixed prostheses. These findings suggest that the bond between zirconia and cement was not always the most susceptible area for failures. For PICN, mixed failures were more frequent in the R group, which may be related to the strong interaction between resin cement and the silanized ceramic surface, composing a monoblock. Catastrophic failures were found in both PICN and 5Y‐PSZ zirconia for the GI and RM‐GI groups. These failures may be related to the nonadhesive properties of glass ionomer, which can increase the adhesive failures, especially in repetitive chewing cycle situations. Consequently, crack propagation may be generated from the cumulative effect of chewing [40, 41] and possibly result in catastrophic failures.

Various test methods, such as macroshear, microshear, macrotensile, and microtensile tests, have been used for evaluating the bond strength of dental ceramics. Regardless of the testing method, it is crucial that the bonding interface is the most stressed region, ensuring accurate measurement of bond strength values [42]. To evaluate the adhesion of 5Y‐PSZ zirconia and PICN, this study initially adopted the microtensile test. The vertical direction of load relative to the ceramic bonding interface and the small size of the microbars tested by this method reduce the chance of structural failures [43]. Although multiple sticks were obtained from the same block, each stick was considered an individual specimen for statistical analysis, as commonly reported in the literature [20, 21, 22]. However, it is acknowledged that this approach may introduce a degree of pseudo‐replication, given that specimens originating from the same block are not entirely independent. This methodological limitation was taken into account in the interpretation of the results. The microtensile results obtained by PICN showed that the mean bond strength in the R and RM‐GI groups was statistically similar and better than in the GI group. Additionally, it can be seen that the R group generated more microbars and apparently is the most consistent among the three tested groups. According to Browning et al. [44], although glass ionomer interacts at the interface with the dental structure creating covalent bonds, the role of these bonds is not significant in increasing retention, which is usually low for reasons such as: spontaneous cohesive fracture of the cement; high stress generated by shrinkage; adhesion restrictions of the cement to the crown; dentin walls or geometric configuration where there are few opportunities for stress relief by plastic deformation or cement flow; or the fact that glass ionomer have rapidly dehydration and contraction in air or moisture.

Hydrofluoric acid tends to dissolve the glass phase of PICN while the polymer network remains intact, creating a structure with high potential for micromechanical interlocking [45]. The application of silane increases surface wettability, facilitating the formation of covalent bonds between the restorative material and resin cement, resulting in effective interaction [46]. In this study, the use of hydrofluoric acid followed by silane in the PICN group likely favored the results observed for this group. Similar findings were previously reported by Hilgemberg et al. [47], who evaluated the effect of different strategies on the microtensile bond strength of cementation agents to CAD/CAM composites and found that the highest values were achieved when silane was applied before the resin cement. They concluded that the effectiveness of the bonding strategy may depend on the composite, resin cement, adhesive, and silane employed.

The present study has limitations that should be addressed in future research, such as the use of artificial dental substrates, which may not fully reflect the complexity of natural teeth, and the in vitro environment, which does not consider the dynamic oral conditions such as humidity, temperature, and salivary contaminants. Further clinical studies are needed to evaluate the long‐term performance of these cementation systems. Regarding the clinical applicability of this study, dentists may prefer resin cements for PICN restorations due to their superior adhesion and compressive strength. For 5Y‐PSZ restorations, the type of cement had less impact on compressive strength, but resin cements still provided better adhesion. The choice of cement should consider the clinical conditions and the expected loading of the restoration.

Resin cements yield superior compressive strength results after aging compared to glass ionomer‐based cements for PICN restorations. For 5Y‐PSZ restorations, the type of cement does not significantly affect compressive strength. Regardless of the cement, the PICN ceramics achieved better compressive strength compared to the 5Y‐PSZ ceramics. Resin cements exhibit superior adhesive performance compared to glass ionomer‐based cements for both 5Y‐PSZ zirconia and PICN ceramics.

## AUTHOR CONTRIBUTIONS

**Conceptualization**: Letícia Moreschi and Pedro Henrique Corazza. **Formal analysis**: Letícia Moreschi and Pedro Henrique Corazza. **Investigation**: João Paulo De Carli, Marciele Cristiane Spanemberg Fuhr, Renan Brandenburg dos Santos, and Paulo Renato Pulga da Silva. **Methodology**: João Paulo De Carli, Marciele Cristiane Spanemberg Fuhr, and Renan Brandenburg dos Santos. **Writing—original draft**: Letícia Moreschi and Pedro Henrique Corazza. **Writing—review & editing**: Letícia Moreschi and Pedro Henrique Corazza.

## CONFLICT OF INTEREST STATEMENT

The authors do not have any financial interest in the companies whose materials are included in this article.
